# Adsorption of Naphthalene on Clay Minerals: A Molecular Dynamics Simulation Study

**DOI:** 10.3390/ma15155120

**Published:** 2022-07-23

**Authors:** Zhixin Chen, Liming Hu

**Affiliations:** State Key Laboratory of Hydro-Science and Engineering, Department of Hydraulic Engineering, Tsinghua University, Beijing 100084, China; czx19@mails.tsinghua.edu.cn

**Keywords:** polycyclic aromatic hydrocarbons (PAHs), clay minerals, adsorption mechanism, molecular dynamics simulation (MD)

## Abstract

Naphthalene, as one of the representative polycyclic aromatic hydrocarbons, widely exists in contaminated sites and is a potential threat to human health due to its high mobility in soil. The interaction between naphthalene and clay minerals is of great significance to the environmental behavior of naphthalene and the design of remediation technology. In this study, montmorillonite and kaolinite were selected as representative clay minerals. Naphthalene adsorption behavior on mineral surfaces and water-wet kaolinite surfaces was investigated using molecular dynamics (MD) simulation. The interaction energy was calculated to represent the interaction between naphthalene and soil fractions, and the relative concentration and density distribution of naphthalene was analyzed to describe the distribution of naphthalene on the clay surfaces. The self-diffusion coefficient of naphthalene was obtained to represent its mobility under different water content. The electron density calculation was performed to reveal the different adsorption behavior of naphthalene on different surfaces of kaolinite. The simulation results show that montmorillonite had a stronger interaction with naphthalene due to larger electrostatic interaction energy compared to kaolinite, and naphthalene distributed more intensively on the montmorillonite surface. With regards to kaolinite, naphthalene tended to be absorbed on the alumina octahedral surface rather than the silicon tetrahedral surface due to the weak hydron bond interaction. The results indicate that water impeded the adsorption of naphthalene, and the optimal initial thickness of water film, which was 10 Å, was put forward for the application of thermal remediation technology. Furthermore, the average interaction energies between water and mineral surfaces largely depended on the water content, and the competitive adsorption between water and naphthalene only occurred under absorbed and bound water conditions. Overall, the knowledge of naphthalene–soil fractions interaction gained in this study is critical to the understanding of the environmental behavior of naphthalene and the reference for remediation technology.

## 1. Introduction

Polycyclic aromatic hydrocarbons (PAHs) are one of the hazardous persistent organic pollutions (POPs) that are ubiquitous in air, soil, and sediments [1,2]. Due to their high potential to threaten human health with mutagenicity, carcinogenicity, and teratogenicity, several remediation technologies, such as thermal desorption, soil washing, etc., have been developed to effectively remove them from soils, which are the major sink to PAHs [3,4]. PAHs are composed of fused benzene rings that contain hydrogen and carbon elements. Their mobility and bioavailability decrease with the increase in molecular weight. According to the advocation of Green Sustainable Remediation (GSR) [5,6], it is more suitable for PAHs with high molecular weight to be controlled through risk management. This is due to the fact that it is difficult to degrade them as they are relatively stable and have a strong interaction with soil minerals [7]. However, PAHs with low molecular weight, such as naphthalene, have high mobility in soils and might transfer through the unsaturated zone and contaminate the groundwater [8,9,10]. Thus, the adsorption/desorption behavior of naphthalene in soils needs to be investigated to develop an efficient remediation technology.

The most significant process that determines the behavior of naphthalene in the subsurface is its sorption in soils [11,12,13]. Adsorption behavior is greatly influenced by soil texture, soil water content, soil organic content, and temperature [8,14,15,16]. Among them, clay minerals, with a larger specific surface area and higher porosity, can strongly bind hydrophobic PAHs compared to sand [12,17,18,19]. Although it has been shown that the contribution of clay minerals to the sorption of PAHs is small compared to their partition into soil organic matter [20,21,22], the soil in the deeper layer always has little content of organic matter [14,23,24]. Therefore, the sorption of PAHs onto clay minerals will play a major part in their fate in the environment and their migration into groundwater [25,26]. 

The adsorption behavior of naphthalene in clay minerals has been studied extensively over the past decades. Ref. [14] studied the effect of clay mineral compositions on its partition using the linear partitioning model and showed that the value of the partitioning parameter increases with the increase in the montmorillonite concentration, which means that montmorillonite plays an important role in the adsorption of naphthalene in soils. Refs. [8,17] conducted a series of adsorption kinetic and isothermal experiments involving bentonite absorbing naphthalene from aqueous solutions. The results show that the pseudo-second-order kinetic model is suitable for the kinetic process, and that the Freundlich isotherm model is suitable for isothermal adsorption results. Several studies have verified that clay minerals interact with hydrophobic organic compounds. Most studies considered the adsorption ability of clay minerals in aqueous solutions [10,27], where the clay minerals were fully hydrated. However, in the unsaturated zone, the water content of clay minerals varies with the humidity of the atmosphere. The reason that the surfaces of the clay minerals have a strong interaction with water molecules is due to the electromagnetic potential [28], which, in turn, influence the absorption behavior of naphthalene. In addition, although the classical sorption model is suitable for experimental data [11], it does not reveal the microscopic mechanism of adsorption behavior. Therefore, it is difficult to predict the adsorption behavior of naphthalene on unsaturated clay minerals.

In the adsorption process of naphthalene onto clay minerals, the mass transfer process mainly involves the accumulation of naphthalene at the interface of two phases (naphthalene–water, naphthalene–minerals). The interactions between the adsorbed naphthalene and water or the surfaces of minerals have a physical nature. These interactions are induced by van der Waals forces and electrostatic forces, and the adsorption process is reversible [12,27]. Molecular dynamic simulation is one kind of computer simulation method that investigates the physical movements of atoms and molecules determined by numerically solving Newton’s equations within a fixed period time. It is a powerful technique for computing the equilibrium and transport properties of a classical many-body system [29]. Recently, molecular dynamics simulation has been recognized as a versatile toolbox to analyze physical mechanisms at the microscopic level. It has been applied to mimic the equilibrium adsorption between minerals and several organic substances to observe the microscopic adsorption process [30,31,32,33]. Ref. [30] used MD to investigate the equilibrium partitioning of different oil fractions on quartz surfaces under different temperature conditions with the calculation of sorption energy. Ref. [34] conducted the MD of benzene adsorption onto montmorillonite to study the influence of hydration status. During their simulation, different water molecules were added to the mineral surfaces to represent different states of hydration. Ref. [35] established the models to analyze the influence of humic acid and water in the adsorption of PAHs on sandy soil, in which the adsorption energy, self-diffusion coefficient, and radial distribution function were used to describe the interaction between PAHs and different soil fractions. The results show that the presence of water has a remarkable negative effect on the adsorption of PAHs on quartz. 

The environmental behavior of naphthalene on clay mineral surfaces is of great significance. This is due to the fact that it is critical for the efficiency of soil remediation technology such as thermal desorption technology, which is the reverse process of absorption [36]. For thermal desorption technology, the water in the soil evaporates during the process of increasing the temperature to above 100 °C, which consumes lots of energy [37]. In addition, if the interaction between naphthalene and clay minerals is weakened by the presence of water, the maximum temperature and heating time is largely reduced. This means that the initial water content of the contaminated soils has a significant impact on the efficiency and energy-consumption of this technology. Although moisture plays a significant role in the interaction between naphthalene and clay minerals, the study of the influence of water content and the corresponding mechanism remains sparse. 

Therefore, a comprehensive understanding of the mechanism of naphthalene–clay interaction will facilitate the understanding of the environmental behavior of naphthalene as well as the development and application of soil remediation technologies based on the desorption process. In this work, the adsorption behavior of naphthalene on clay minerals was investigated at the molecular level using MD. Interaction energy, relative concentration, self-diffusion coefficient, and electron density difference were used to describe the interaction between naphthalene and clay minerals. There were three objectives of this work: (i) to analyze the adsorption potential between naphthalene and two representative clay minerals, kaolinite and montmorillonite; (ii) to investigate the effect of water content on the interaction between naphthalene and kaolinite; and (iii) to examine the competitive adsorption between water and naphthalene under different water conditions.

## 2. Theoretical Models and Methods

### 2.1. Model Construction

A range of software can be used to run MD simulations, such as LAMMPS, CHRMM, and Materials Studio. Among them, Materials Studio is a commercial software that has a friendly interface and modern nonbond calculation methods. The models built by Materials Studio are run by high-performance computing with a high efficiency of calculation [38].

In this study, MD simulation was carried out by means of Materials Studio of Accelrys Inc. The built models were composed of naphthalene layers, aqueous layers with different thicknesses, and mineral surfaces.

With regards to the mineral surfaces, the initial unit cells were taken from the Surface Model Database V1.5 of INTERFACE [39]. Repeat units of kaolinite were cleaved along (001) and (00-1) crystallographic orientation to create the silicon tetrahedral surface and the alumina octahedral surface, respectively. Next, supercells of 10 × 6 × 2 were rebuilt with dimensions of 5.154 nm × 5.365 nm × 1.478 nm to form the kaolinite surface layers. A montmorillonite supercell of 10 × 6 × 2 was created by the same method with dimensions of 5.191 nm × 5.409 nm × 2.005 nm, which had an optimized Al^3+^ with Mg^2+^ substitution pattern and K^+^ interlayer cations. The lattice parameters of the unit cell are shown in Table 1. The cation exchange capacity (CEC) of kaolinite and montmorillonite was 0 mmol/100 g and 90 mmol/100 g, respectively. With regards to the naphthalene layer, 30 naphthalene molecules were placed into a box with the same surface dimensions as the respective clay minerals. With regards to the aqueous layer, different water molecules were also placed into a box that had the same surface dimension as the clay minerals to form different thicknesses of the water film. 

**Figure 1:**
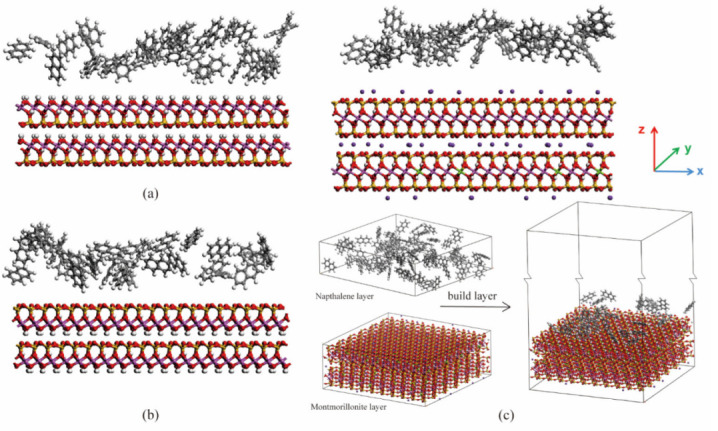
The initial configuration of the binary model for (**a**) kaolinite (aluminum octahedral surface); (**b**) kaolinite (silicon tetrahedral surface); (**c**) montmorillonite.

**Figure 2:**
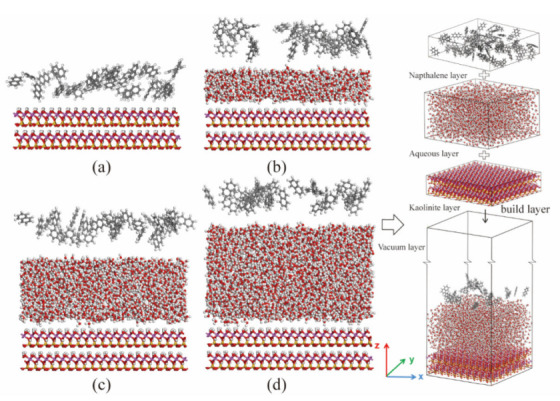
The initial configuration of the ternary model with different thicknesses of the water film. (**a**) 0 Å; (**b**) 10 Å; (**c**) 20 Å; (**d**) 30 Å.

In our research, two kinds of models were taken into consideration: binary models, and ternary models, as shown in Figure 1 and Figure 2. The montmorillonite structure contains silicon (yellow), oxygen (red), aluminum (pink), magnesium (green), potassium (purple), and hydrogen (white) atoms. The kaolinite structure contains the same types of atoms as montmorillonite except for magnesium. The naphthalene molecules contain carbon (grey) and hydrogen (white) atoms. In Figure 2, water molecules contain oxygen (red) and hydrogen (white) atoms. As kaolinite is composed of a tetrahedral sheet and an octahedral sheet, two structures were considered in binary models: the silicon tetrahedral surface–naphthalene model, and the alumina octahedral surface–naphthalene model. The binary models were developed by placing the naphthalene layer on different mineral surfaces, as shown in Figure 1c. The ternary models were created by orderly adding the aqueous layer and naphthalene layer to the surface of clay minerals, as seen in Figure 2d. To avoid the influence of the upper atoms due to the periodic boundary condition, a vacuum layer of 10 nm was added to all the created models. 

### 2.2. Molecular Dynamics Simulation

In MD simulation, the thermodynamically consistent force field (INTERFACE-PCFF) for the assembly of inorganic and organic nanostructure was adopted [39]. The canonical ensemble (NVT) was performed at 298 K for all models. The van der Waals energy and electrostatic energy were both calculated using the Ewald summation method [40], which is more costly but is accurate for the calculation of long-range electrostatic interaction [31]. To verify the consistency of the force field, all the molecules in these models were free, which means that no constrain was applied to the atoms in these models. The total simulation time was 1 nanosecond (ns) to thoroughly relax the system, with the time step of 1 femtosecond (fs). All the trajectories were saved, and the frames were output for analysis every 50 fs. The trajectories of the last 200 picoseconds (ps) were adopted for the analysis of the equilibrium condition as the temperature fluctuation was below 5%. Therefore, the interaction energies were obtained by calculating the average value of the interaction energies of the last 200 ps.

### 2.3. Data Analysis

#### 2.3.1. Interaction Energy between Different Layers

The adsorption potential between different layers is quantified by adsorption energy, which can be used as an indicator of the interaction strength between them. Thus, the adsorption energy has an equivalence with the interaction energy between two layers, and is calculated using the following equation:

(1) $$E_{i/j}=(E_{i}+E_{j})-E_{\mathrm{total}}$$

where *E_i_*_/*j*_ (*i* ≠ *j*) is the interaction energy between layer *i* and layer *j*; *E_i_* and *E_j_* represent the energy of layer *i* and layer *j*, respectively; and *E*_total_ denotes the total energy of the system consisting of both layer *i* and layer *j*. For the ternary models, the remaining layer was removed during the analysis of the interaction energy between the two selected layers.

The energy *E* is composed of van der Waals energy (*E*_vdw_) and electrostatic energy (*E*_ele_), which is nonbonding interaction energy. The calculation of energy *E* is written as:

(2) $$E=E_{\mathrm{vdw}}+E_{\mathrm{ele}}=\sum_{ij}\varepsilon_{0,ij}\left[2{\left(\frac{\sigma_{0,ij}}{r_{ij}}\right)}^{9}-3{\left(\frac{\sigma_{0,ij}}{r_{ij}}\right)}^{6}\right]+\frac{1}{4\pi \varepsilon_{0}}\sum_{ij}\frac{q_{i}q_{j}}{r_{ij}}$$

where *σ*_0,*ij*_ is the equilibrium nonbonding distance between two atoms, *ε*_0,*ij*_ represents the energy at the equilibrium distance, and *r_ij_* is the distance between two atoms. Both *σ*_0,*ij*_ and *ε*_0,*ij*_ are gained by standard combination rules [33,39]. *q_i_* and *q_j_* are the partial charges of atom *i* and *j*, and *ε*_0_ is the dielectric permittivity of the vacuum.

Negative interaction energy means attractive force between the two components, while a positive value of adsorption energy represents repulsive force. Therefore, the larger the absolute value of the negative adsorption value, the more intensive the interaction strength, which means that it is more difficult to desorb contaminants from the clay mineral surface. In this study, the results show that naphthalene had interactions with different soil fractions; therefore, the value of the interaction energies was always negative. For convenience, the absolute value of these energies was used for further discussion. 

#### 2.3.2. Self-Diffusion Coefficient of Naphthalene

Mean square displacement (*MSD*) is obtained from the particle positions in a MD simulation. It reflects the deviation of the spatial position of molecules from the initial state to the equilibrium state, which is identified as follows:

(3) $$MSD(\Delta t)=\frac{1}{\tau -\Delta t}\int_{0}^{\tau -\Delta t}{\left|r_{i}(t-\Delta t)-r_{i}(t)\right|}^{2}dt=\langle {\left|r_{i}(t-\Delta t)-r_{i}(t)\right|}^{2}\rangle$$

where *r_i_*(*t*) is the position of particle *i* at the time *t*, Δ*t* is the interval of time, and *τ* is the simulation time.

The self-diffusion coefficient *D* is described by Equation (4), which is calculated by fitting the slope of *MSD* and dividing by 6, according to Einstein’s diffusion law [41]. The value of *D* is used to investigate the moving velocity of the specific molecules. It reflects the dynamic properties of naphthalene on mineral surfaces.

(4) $$D=\frac{1}{6}\underset{t\to \infty }{\lim}\frac{dMSD}{dt}$$

#### 2.3.3. Density Functional Theory (DFT) Calculation

The electron density difference calculations were conducted to reveal the electron density change when the naphthalene molecule is absorbed on the surface of clay minerals. These reflect the different adsorption mechanisms of naphthalene on the tetrahedral surface and octahedral surface of the kaolinite, which are defined as follows:

(5) $$\Delta \rho (r)=\rho_{\mathrm{surf}-\mathrm{nap}}(r)-\rho_{\mathrm{surf}}(r)-\rho_{\mathrm{nap}}(r)$$

where *ρ*_surf-nap_(**r**) is the electron density of the naphthalene-absorbed surface, and *ρ*_surf_(**r**) and *ρ*_nap_(**r**) are the electron density of the surface and naphthalene, respectively [42].

To reduce the computational cost, binary models with smaller surface dimensions were built. Each contained a kaolinite layer with dimensions of 1.546 nm × 1.788 nm × 0.739 nm and a naphthalene molecule that was put on the surface of the kaolinite. Geometry optimization was performed on this structure to obtain the optimized configuration of naphthalene on the kaolinite surface using the lowest energy. All the relative calculations were performed byDFT calculations. The valence electron density has been expanded in a plane-wave basis set with a cutoff of 571.4 eV.

## 3. Results and Discussions

### 3.1. Effect of Surface of Different Minerals 

**Figure 3:**
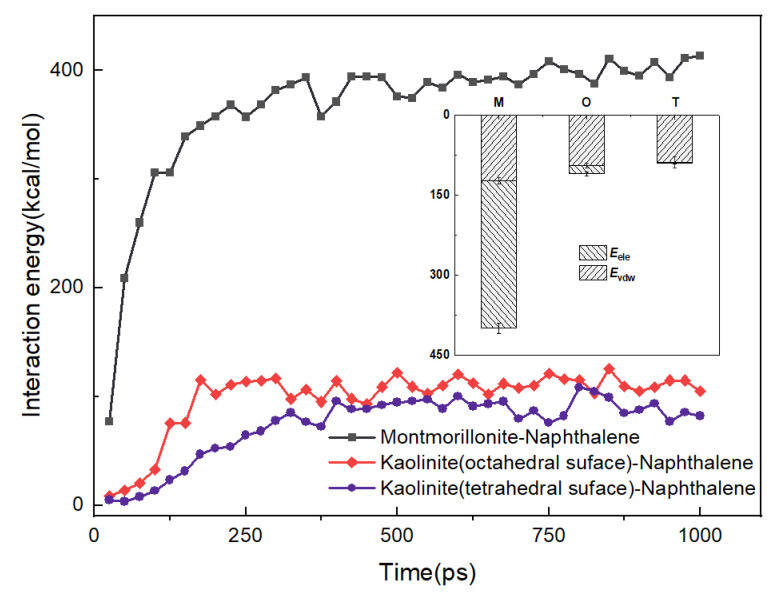
The interaction energies between montmorillonite and kaolinite surfaces and the naphthalene molecules in relation to the change in simulation time.

**Figure 4:**
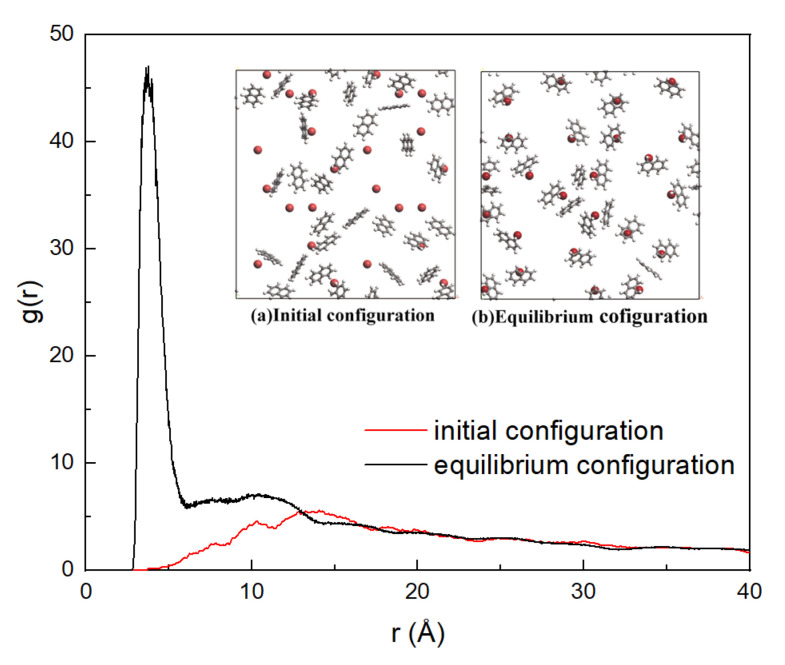
The RFD of potassium atoms (red ball) with naphthalene. The inset figures are (**a**) the initial configuration of naphthalene absorbed on the montmorillonite surface and (**b**) the equilibrium configuration of naphthalene absorbed on the montmorillonite surface.

**Figure 5:**
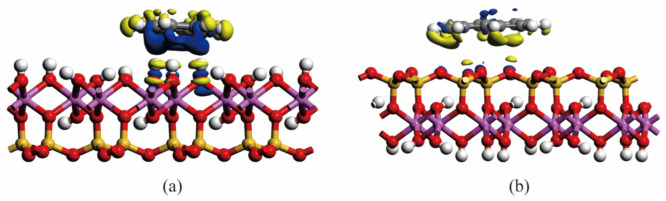
Electron density difference of (**a**) alumina octahedral surface–naphthalene, (**b**) silicon tetrahedral surface–naphthalene.

As mentioned in Section 2.1, the binary models were developed to investigate the adsorption of naphthalene on different natural mineral surfaces. The interaction energies between naphthalene and different minerals can be seen in Figure 3. The kaolinite surfaces contain the octahedral surface and the tetrahedral surface. The inset is the two components of interaction energy including van der Waals energy (*E*_vdw_) and electrostatic energy (*E*_ele_). M, O, and T refer to the interaction energies of naphthalene with montmorillonite, octahedral surface, and tetrahedral surface, respectively. At the initial stage, although the naphthalene molecules were distributed on the mineral surfaces after the geometry optimization, the interaction energies were quite small. As the MD process occurred, the interaction energies between naphthalene and minerals increased, which meant that the naphthalene molecules were adsorbed on the mineral surfaces. As shown in Figure 3, the interaction energies reached an equilibrium state at the end of the simulations. The average interaction energies during the last 200 ps between naphthalene and mineral surfaces including montmorillonite, the alumina octahedral surface, and silica tetrahedral surface were 401.76 kcal/mol, 111.36 kcal/mol, and 91.49 kcal/mol, respectively. The contribution of van der Waals energy (*E*_vdw_) and electrostatic energy (*E*_ele_) is demonstrated in the inset of Figure 3. It is obvious that the van der Waals energies between naphthalene and different minerals are quite similar, while the electrostatic energies show a great difference. The electrostatic energy with montmorillonite is slightly larger than with kaolinite. The montmorillonite mineral has isomorphous substitution, so there are some cations distributed on the surface [43] which contribute to the electrostatic interaction energy between contaminants and the clay minerals. Therefore, the radial distribution function of potassium cations with naphthalene was calculated to describe the interaction between cations and naphthalene. Figure 4 shows that the distance distribution possibility between potassium cations and naphthalene largely increased from the initial to the equilibrium configuration, which can also be seen in the inset figure in Figure 4. To see the relative location between potassium atoms and naphthalene clearly, only the potassium atoms on the surface of the clay structure are displayed. Although naphthalene is one kind of nonpolar organic compound, it has an aromatic π system which can result in a noncovalent cation–π interaction between a cation and the planar surface of naphthalene [44]. This mechanism accounted for the phenomenon that montmorillonite had a stronger binding with PAHs than kaolinite, which has been observed in a previous experiment [14]. With regards to kaolinite, the interaction energy with the alumina octahedral surface was larger than that with the silicon tetrahedral surface. Naphthalene tended to be absorbed on the surface of the alumina octahedral surface due to the increase in electrostatic interaction energy. The electron density difference of the naphthalene-absorbed kaolinite surface is depicted in Figure 5. Both the octahedral and tetrahedral surfaces were considered. The difference indicates the reorganization of electronic density caused by adsorption. The yellow region represents the decrease in electron density, while the blue region indicates the increase in electron density. The iso-surface values of Figure 5a are +0.005 and −0.005 e/Å^3^. The iso-surface values of Figure 5b are +0.0025 and −0.0025 e/Å^3^. As seen in Figure 5, the electron density difference of the octahedral surface was larger than the alumina surface, which means that the interaction between naphthalene and the alumina surface with the hydroxyl surface was more evident. In Figure 5a, it can be seen that the electron density around the aromatic ring increased, while the electron density around the hydrogen atoms decreased. This is due to the formation of OH–π interaction between naphthalene and the alumina octahedral surface. Ref [45] also found that the π electronic cloud of phenanthrene forms OH–π bonds with the alumina surface of kaolinite, which belongs to weak hydrogen bonds. Therefore, as weak hydron bonds are one kind of electrostatic interaction [46], it can be concluded that the octahedral surface had a stronger interaction with naphthalene than the tetrahedral surface because of the formation of weak hydron bonds.

**Figure 6:**
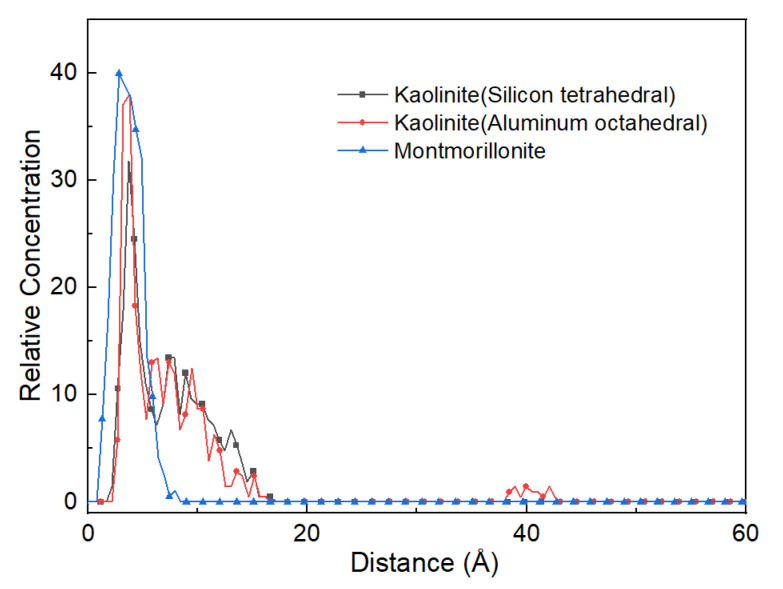
The relative concentration of naphthalene on different mineral surfaces.

**Figure 7:**
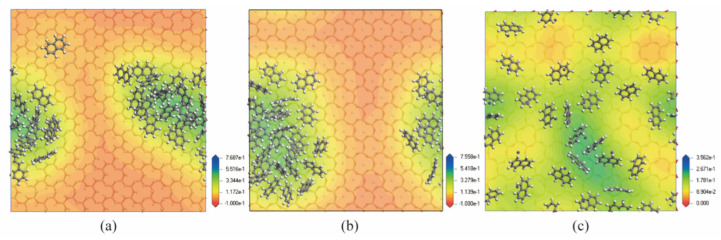
The equilibrium configuration and density distribution of naphthalene on different mineral surfaces. (**a**) Kaolinite (Aluminum octahedral surface); (**b**) Kaolinite (Silicon tetrahedral surface); (**c**) Montmorillonite.

Furthermore, to reveal the distribution of naphthalene on mineral surfaces, the relative concentration profiles along the z-axis were calculated. The equilibrium configuration and density distribution of the naphthalene on mineral surfaces were also obtained. As Figure 6 and Figure 7 show, the naphthalene molecules were closer to the montmorillonite surfaces than the kaolinite surfaces. All the naphthalene molecules were also intensively scattered on the surface of montmorillonite. While the distribution conditions of naphthalene on kaolinite surfaces were nearly similar, most molecules existed in the distance range of 5~20 Å. The patterns of the relative concentration of naphthalene on the surfaces of clay minerals were coincident with the interaction energies among them.

### 3.2. Effect of Moisture Content

Therefore, with the comparison of interaction energy, relative concentration, and density distribution, it was concluded that the adsorption potential for naphthalene followed the order of montmorillonite > alumina octahedral surface > silicon tetrahedral surface.

Although the adsorption potential of naphthalene on montmorillonite was larger than that of kaolinite, the former constitutes a far smaller fraction of the total soil mineral content compared to aluminosilicate kaolinite minerals [47,48]. Moreover, it has been mentioned in Section 3.1 that naphthalene was inclined to be adsorbed onto the alumina octahedral sheet. Therefore, the adsorption mechanism of naphthalene on the alumina octahedral surface of kaolinite under different water content was studied. The weight water content of the molecular model is calculated using the following equation:

(6) $$w=SSA\times H\times \rho_{w}$$

where *SSA* is the specific surface area of minerals, *H* is the thickness of the water film, and *ρ_w_* is the density of water. The relationship between water content and thickness of water film is shown in Table 2. 

**Figure 8:**
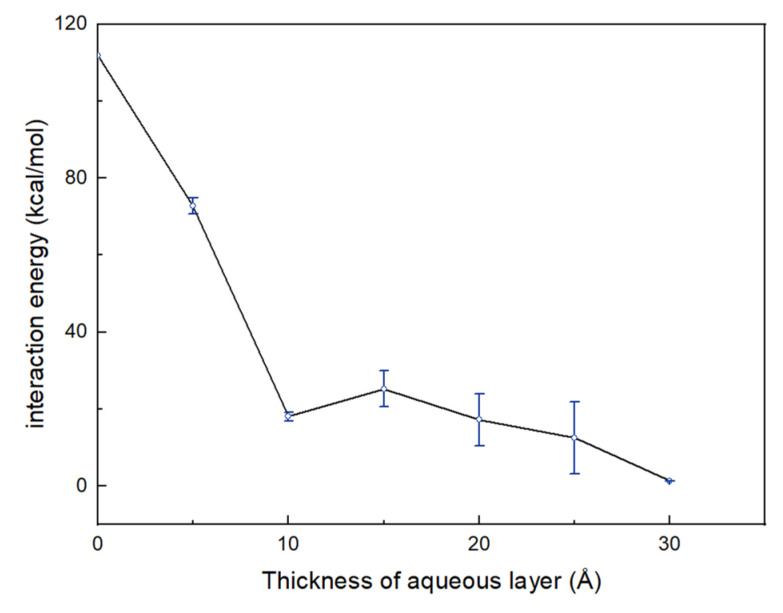
The interaction energy between naphthalene and kaolinite (alumina octahedral surface) under the different thicknesses of an aqueous layer.

In this research, the specific surface area of kaolinite was assigned as 20 m^2^/g, which was based on experiment results [27]. Six water films with different thicknesses from 5 Å to 30 Å with an interval of 5 Å were considered to investigate the effect of moisture content. Some are shown in Figure 2. Furthermore, to reveal the impact of naphthalene on the soil–water system, the models that contained only water and minerals were established as the blank control group for the ternary model. To avoid randomness, each of the ternary models was run three times. As shown in Figure 8, the interaction energies between naphthalene and kaolinite (the alumina octahedral surface) changed with the increase in the aqueous layer. Before the thickness of the water film reached 10 Å, the energy decreased sharply, and when the thickness was larger than 10 Å, the energy increased slightly and then decreased to nearly zero. This tendency shows that water played an important part in the adsorption of naphthalene. Due to the hydrophilicity of the mineral surface, polar water molecules are absorbed onto the surface to create a water film covering the clay particle surface [49]. This impedes the adsorption of naphthalene onto the surface because the distance between the mineral surface and naphthalene increases. However, as the amount of the water molecules increased, naphthalene was able to slightly diffuse into the aqueous layer, enhancing the adsorption of naphthalene. Ref [24] found that the solubility of SVOCs (semi-volatile organic contaminants) is significant for the behavior of SVOCs in soils. However, since the solubility of naphthalene is quite small in water (31.7 mg/L at 25 °C), this diffusion effect barely changes the adsorption of naphthalene, which may explain the slight increase in the adsorption energy between naphthalene and clay mineral. In the physical or thermal remediation technologies for contaminated sites, the removal efficiency highly depends on the interaction between contaminants and soil fractions [50,51,52]. In addition, it has been concluded that the existence of water caused a lot of extra energy consumption when thermal desorption techniques were applied [51]. Therefore, there may exist an optimal initial water content for thermal desorption remediation that may not only decrease the adsorption between naphthalene and octahedral surfaces but also save energy used for the evaporation of water. This means that the analysis of the interaction between naphthalene and clay mineral may be an indicator of energy consumption when thermal desorption technology is applied.

**Figure 9:**
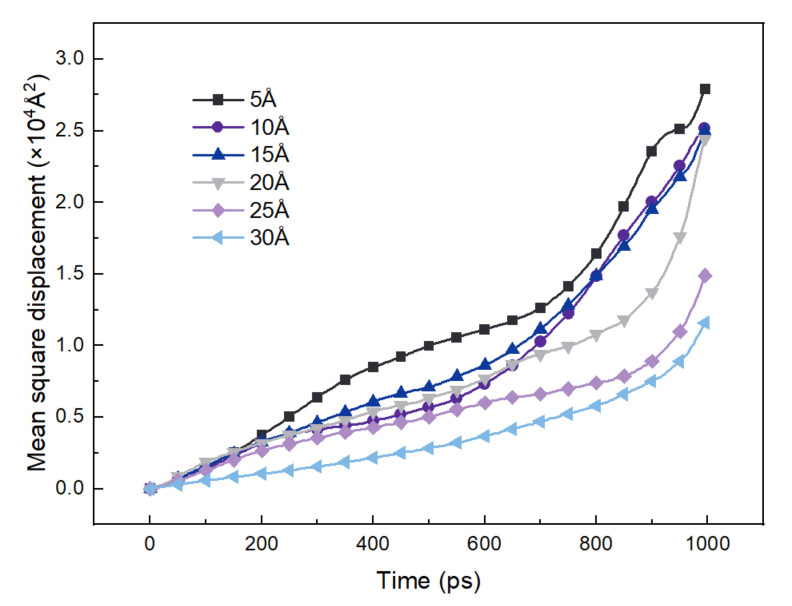
*MSD* of naphthalene on kaolinite (alumina octahedral surface)/water interface.

**Figure 10:**
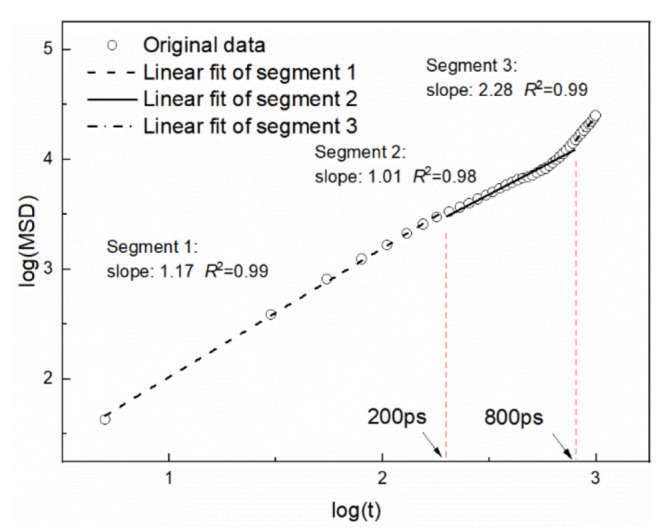
MSD of naphthalene on kaolinite (alumina octahedral surface)/water interface (log(*MSD*) vs. log(*t*)) under the water film of 15 Å.

In the adsorption process, the mobility of naphthalene on the kaolinite (alumina octahedral surface)/water surface is important. Therefore, the *MSD* of naphthalene was counted during the dynamic process, as shown in Figure 9. Anomalous diffusion may exist that has an impact on the pathway of naphthalene that cannot be ignored. Therefore, the slope of log(*MSD*) ~ log(*t*) was calculated to judge if Einstein diffusion occurred [30]. If the slope is not 1, it means that it is impossible to use the MD trajectories for calculating *D*. An example of the water film of 15 Å is shown in Figure 10. The slope of the log(*MSD*) ~ log(*t*) was 1.17 before 200 ps indicating that anomalous diffusion occurred at the beginning of the MD. The slope was up to 2.28 after 800 ps which indicated the noise portion. Therefore, the self-diffusion coefficients were determined according to the trajectories between 200 ps and 800 ps and are shown in Table 3. It is evident that there was a negative correlation between the interaction energies and self-diffusion coefficients. This means that the stronger the interaction between naphthalene and the hydrated kaolinite surface, the larger the mobility of naphthalene to make adsorption occur.

### 3.3. Competitive Adsorption between Naphthalene and Water

**Figure 11:**
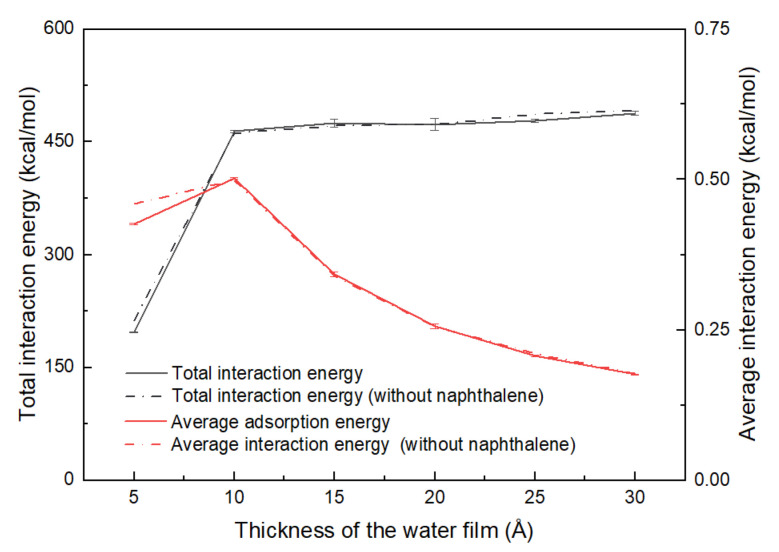
The total interaction energies and average interaction energies between the water molecules and kaolinite (alumina octahedral surface) under the different thicknesses of the aqueous layer.

It has been reported that organic substances may have competitive adsorption with water, which is reflected in the change in interaction energy [31]. For comparison, the models without naphthalene and the ternary model with naphthalene were created. The total interaction energy and average interaction energy between water and clay minerals of these two kinds of models were calculated. Figure 11 shows that with the increase in the thickness of the water film, the total interaction energy between water and the mineral surface increased quickly at first, and then reached a relatively stable platform with a slight increment. The value of the total interaction energy was above 460 kcal/mol. It proved that there was an intensive interaction between the clay particle surface and water. This is due to the fact that the alumina octahedral surface is hydrophilic, and water can have a strong interaction with the hydroxyl groups [49]. With regards to the average interaction energies, they were attained by using the total interaction energies divided by the relative number of water molecules. The average interaction energy increased slightly at first and then decreased gradually as the thickness of the water film increased. This pattern corresponds with the concept that there are several water conditions in the clay, including absorbed water, bound water, capillary water, and gravitational water [24,53,54]. The absorbed water and bound water have larger interactions with the kaolinite surface; thus, the average interaction energy of each water molecule is higher. Ref [24] pointed out that the water content of bound water in kaolinite is above 2%, which corresponds with the thickness of 10 Å in this study. Therefore, when the thickness was over 10 Å, some capillary water would be present, and the average energies would decrease. From this point of view, the rationality of these models can be proved. 

**Figure 12:**
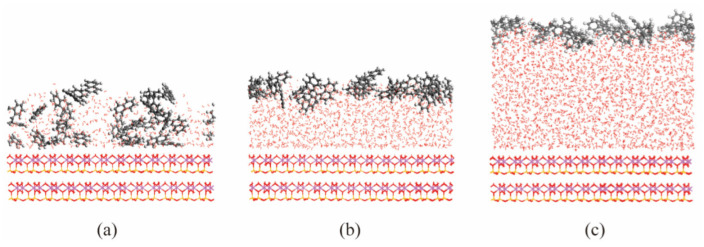
The equilibrium configuration of ternary models under the different thicknesses of the aqueous layer. (**a**) 5 Å; (**b**) 10 Å; (**c**) 15 Å.

As shown in Figure 11, the interaction energies of these two models had a large difference only at the thickness of water film of 5 Å, and the energies of models without naphthalene were higher than the energy of the ternary model. This means that naphthalene only had competitive adsorption with water molecules when the water film was thin, so that the interaction energy between water and clay mineral was reduced. This is due to the fact that when the water film was thin, the naphthalene molecules penetrated the water film and occupied the adsorption location on the surface of kaolinite, as shown in Figure 12. If the water content was high enough to form capillary water, it was difficult for the naphthalene molecules to penetrate the water film, and thus, had little effect on the interaction between water and kaolinite.

## 4. Summary and Conclusions

In this study, MD simulation was performed to investigate the molecular behavior of naphthalene on different clay mineral surfaces under different moisture contents. The interaction energy, relative concentration, self-diffusion coefficient, and electron density difference were obtained to represent the interaction between naphthalene and clay minerals.

The results indicate that montmorillonite had a higher potential to adsorb naphthalene molecules than kaolinite because of the larger electrostatic interaction energy, and the naphthalene molecules scattered more intensively on the montmorillonite surface according to the analysis of the relative concentration of naphthalene. With regards to kaolinite, naphthalene molecules tended to be absorbed on the alumina octahedral surface, while the relative concentration and density distribution of naphthalene on both the tetrahedral and octahedral surfaces were quite similar. 

The results also show that water impeded the adsorption of naphthalene on the alumina octahedral surface. This was reflected by the interaction energies and self-diffusion coefficient. There was a critical initial water content of soil for thermal desorption remediation technology that facilitates the desorption of naphthalene and requires less energy for water evaporation. The value of the critical thickness of the water film was 10 Å. 

In addition, the results of calculations show that water had different average adsorption energy with the kaolinite surface under different water content, which was consistent with the water conditions. Naphthalene had competitive adsorption with water molecules under a small thickness of water, where only adsorbed water or bound water was present.

The present study suggests the importance of clay minerals on the adsorption of naphthalene at the molecular level. The MD approach enhances the understanding of the environmental behavior of naphthalene, which is critical for the calculation of energy consumption of remediation technologies such as thermal remediation technology, whose remediation efficiency is highly dependent on the interaction between contaminants and soil fractions.

## Figures and Tables

**Table 1:** Lattice parameters of the unit cell of clay minerals.

Parameter	a/Å	b/Å	c/Å	α/°	β/°	γ/°
Kaolinite	5.154	8.942	7.391	91.930	105.050	89.800
Montmorillonite	5.192	9.015	10.023	90.000	95.735	90.000

**Table 2:** The water content of kaolinite according to the thickness of the water film.

The thickness of water film	5 Å	10 Å	15 Å	20 Å	25 Å	30 Å
Water content/%	1	2	3	4	5	6

**Table 3:** Interaction energy (*E*_1/2_) and self-diffusion coefficients (*D*) of naphthalene on kaolinite/water surface.

The thickness of water film	5 Å	10 Å	15 Å	20 Å	25 Å	30 Å
*E*_1/2_ (kcal/mol)	71.61	18.75	28.03	13.39	7.13	1.37
*D* (10^−8^ m^2^/s)	2.898	2.461	2.842	2.097	1.304	1.303
